# Inhibitory Effects of Sulfate and Nitrate Reduction on Reductive Dechlorination of PCP in a Flooded Paddy Soil

**DOI:** 10.3389/fmicb.2018.00567

**Published:** 2018-03-28

**Authors:** Yan Xu, Lili Xue, Qi Ye, Ashley E. Franks, Min Zhu, Xi Feng, Jianming Xu, Yan He

**Affiliations:** ^1^Institute of Soil and Water Resources and Environmental Science, College of Environmental and Resource Sciences, Zhejiang University, Hangzhou, China; ^2^Zhejiang Provincial Key Laboratory of Agricultural Resources and Environment, Hangzhou, China; ^3^Department of Physiology, Anatomy and Microbiology, School of Life Sciences, La Trobe University, Melbourne, VIC, Australia; ^4^Centre for Future Landscapes, La Trobe University, Melbourne, VIC, Australia

**Keywords:** microbial community, nitrate, pentachlorophenol (PCP), reductive dechlorination, redox processes, sulfate

## Abstract

Pentachlorophenol (PCP) is highly toxic and persistent in soils. Bioreduction of PCP often co-occurs with varying concentrations of sulfate and nitrate in flooded paddy soils where each can act as an electron acceptor. Anaerobic soil microcosms were constructed to evaluate the influence of sulfate and nitrate amendments and their redox processes. Microcosms with varying sulfate and nitrate concentrations demonstrated an inhibitory effect on reductive dechlorination of PCP compared to an untreated control. Compared to nitrate, sulfate exhibited a more significant impact on PCP dechlorination, as evidenced by a lower maximum reaction rate and a longer time to reach the maximum reaction rate. Dechlorination of PCP was initiated at the *ortho-*position, and then at the *para-* and *meta-*positions to form 3-CP as the final product in all microcosms. Deep sequencing of microbial communities in the microcosms revealed a strong variation in bacterial taxon among treatments. Specialized microbial groups, such as the genus of *Desulfovibrio* responding to the addition of sulfate, had a potential to mediate the competitive microbial dechlorination of PCP. Our results provide an insight into the competitive microbial-mediated reductive dechlorination of PCP in natural flooded soil or sediment environments.

## Introduction

Pentachlorophenol (PCP) was widely used as a pesticide (e.g., herbicide and insecticide) and wood preservatives due to a broad-spectrum bactericidal activity (Olaniran and Igbinosa, 2011). Long-term exposure of humans and animals to low levels of PCP can cause damage to the liver, kidneys, blood plasma, and the nervous system (Rodenburg et al., 2010; Hiebl et al., 2011). While the use of PCP was banned in 2015 by 90 signatories to the Stockholm Convention (United Nations Environment Programme, 2015), PCP is a persistent organic pollutant and will remain a widespread problem in wetlands, aquatic environments, and soils for the immediate future (Vallecillo et al., 1999; Hong et al., 2005; Persson et al., 2007).

In the environment, PCP can be degraded through chemical, microbiological, and photochemical processes (Choudhury et al., 1986; Guemiza et al., 2017). Microbial anaerobic reductive dechlorination is considered as an environmentally friendly and low-cost method for bioremediation of soil under water logged conditions (Mcallister et al., 1996; Field and Sierra-Alvarez, 2008). Understanding the anaerobic degradation of PCP and environmental drivers which affect biodegradation is essential to improve the overall bioremediation process (Mcallister et al., 1996; Field and Sierra-Alvarez, 2008; Bosso and Cristinzio, 2014).

Under anaerobic environment, the global geochemical cycles of many elements are driven by redox processes that mediated by microorganisms in the soil environment (Dassonville et al., 2004; Kenwell et al., 2016). Oxidized compounds such as ${NO}_{3}^{-}$, Fe(III) minerals, and ${SO}_{4}^{2-}$ often serve as terminal electron acceptors during microbial anaerobic respiration in soils (Adriaens et al., 1996; Lovley and Coates, 2000). In theory, electron acceptors utilized by anaerobes abided to a thermodynamically determined order, which is known as the microbial redox “tower” (Borch et al., 2010; Chen et al., 2017). Under the redox tower, ${NO}_{3}^{-}$ is reduced by denitrifying bacteria first, followed by reduction of manganese and iron oxides. Then, sulfate reducers covert ${SO}_{4}^{2-}$ to sulfide and finally methanogenesis occurs. The succession of reduction potentials gives rise to a functional and metabolic diversity which moderates the rate of key biogeochemical transformation processes under anaerobic condition.

Reductive dechlorination is one of the most important degradation processes for PCP removal under anaerobic conditions. Since PCP acts as an electron acceptor, dechlorination is expected to be a competitive process with the various electron acceptors that coexist simultaneously in natural soil systems. Many studies have demonstrated that Fe(III) reduction is able to promote reductive dechlorination (Li et al., 2008; Wu et al., 2010; Cao et al., 2012) including our previous studies (Xu et al., 2014, 2015; Xue et al., 2017). In mangrove sediments, dechlorination of PCP significantly suppressed the growth of ${SO}_{4}^{2-}$ reducers, which, in turn, facilitated the production of CH_4_ by diversion of electrons from ${SO}_{4}^{2-}$ reduction to methanogenesis (Xu et al., 2015). The coupling of PCP, Fe(III), ${SO}_{4}^{2-}$ reduction, and CH_4_ production has important implications for microbial community function in contaminated soils.

The presence of both nitrate and sulfate can also influence the reductive dechlorination of PCP in anaerobic soils. Low concentrations of nitrate (<1 mM) can promote iron reduction and reductive dechlorination, due to nitrate acting as nutrients; while higher concentrations of nitrate (between 1 and 30 mM) inhibited the dechlorination of PCP (Yu et al., 2014). Inconsistent effects on dechlorination due to the presence of sulfate have also been reported previously. For example, sulfate reduction has been reported to inhibit the anaerobic degradation of chlorophenol due to competitive exclusion (Alder et al., 1993). In contrast, sulfate in the presence of lactate facilitated dechlorination of PCP (Yang et al., 2009).

To date, while several studies have examined the biodegradation of PCP under different redox conditions in anaerobic environments, inconsistent results have been reported due to incongruent conditions of different studies. Aside from dynamic processes (Yoshida et al., 2007; Bosso and Cristinzio, 2014), few studies have investigated the competitive reductive dechlorination of PCP co-occurring in the presence of different electron acceptors at a community level. The recent development of Nextgen sequencing provides the ability to investigate microbial community-mediated responses that underpin the competitive reductive dechlorination of PCP co-occurring with the reduction of different electron acceptors in natural complex soil environment.

In this study, ${NO}_{3}^{-}$ and ${SO}_{4}^{2-}$ were chose as two classical electron acceptors to investigate functional microbial-mediated competitive relationship between PCP dechlorination and soil redox processes. Anaerobic soil microcosms were constructed with the addition of sulfate and nitrate in varying concentrations and the microbial communities were studied in depth through 16S rRNA amplicon sequencing. We hypothesized that (1) addition of varying amounts of competitive electron acceptors, in our case sulfate and nitrate, would vary PCP dechlorination rates due to their relative different thermodynamic potential in regards to the redox tower and (2) under the increasing sulfate and nitrate reducing conditions, microbial community structures would be enriched with specific microbial functional groups underpinning the changed microbial-mediated competitive dechlorination of PCP.

## Materials and Methods

### Soils

Paddy soil samples used in this study were collected from Jiaxing, Zhejiang province (30°50’8.74″ N, 120°43’3.68″ E), China. Soil was sampled from the surface (0–20 cm) and was free of detectable PCP or its dechlorinated products. In order to produce replicate homogenized samples, soils were air-dried and passed through a 2 mm mesh sieve and stored at 4°C prior to the batch experiments (Chen et al., 2014). The basic physicochemical properties of the soil were as follows: pH (6.6), ${NO}_{3}^{-}$ (0.3 mg kg^-1^), ${NH}_{4}^{+}$ (11.5 mg kg^-1^), ${SO}_{4}^{2-}$ (640.9 mg kg^-1^), and free Fe (278.3 mg kg^-1^).

### Chemicals

Pentachlorophenol (≥98% purity), standard solution containing 2,3,4,5-tetrachlorophenol (TeCP), 3,4,5-trichlorophenol (TCP), 3,5-dichlorophenol (DCP) and 3-chlorophenol (3-CP) (≥99.9% HPLC purity), and Na_2_MoO_4_ (99.0%, AR) were all purchased from Sigma-Aldrich (St. Louis, MO, United States). NaNO_3_ (99.0%, AR) and Na_2_SO_4_ (99.0%, AR) were purchased from Sinopharm Chemical Reagent Co., Ltd., China.

### Soil Microcosms

Anaerobic incubation experiments were conducted in microcosms housed in 120 ml serum bottles with crimp sealed aters-coated butyl rubber stoppers (Chunbo, China). All serum bottles, butyl rubber stoppers, and water were sterilized by autoclave at 121°C for 20 min before use. Microcosms contained 15.0 g soil (dry weight), 20 mM lactate, and varying concentrations of sulfate and nitrate in a final 1:1 (w/v) soil/water mixture in the serum bottles. Biological, sterile, and sulfate-reducing inhibited controls were created through the use of soil and lactate without addition of sulfate or nitrate, the use of gamma-radiated soil (50k Gy γ-ray sterilization) and the addition of 20 mM Na_2_MoO_4_, respectively. PCP that dissolved in methanol (1‰, v/v) solvent was added to each microcosm to a final concentration of 150 μM and dried for 24 h to remove methanol before incubation. Microcosms were uniformly mixed and purged under N_2_ stream for 20 min to remove oxygen before being sealed with aters-coated butyl rubber stopper and crimp sealed, as previously described (Xu et al., 2015). The microcosms were then placed in an anaerobic chamber (Electrotex AW200SG, England) and incubated in the dark at 30°C. All treatments were conducted in triplicate and included: (1) the sterile control, (2) control without addition, (3) 20 mM Na_2_MoO_4_, (4) 5 mM Na_2_SO_4_, (5) 20 mM Na_2_SO_4_, (6) 5 mM NaNO_3_, and (7) 10 mM NaNO_3_. Treatments were sampled at days 0, 3 (only for ${NO}_{3}^{-}$ detection), 7, 12, 17, 22, and 40.

### Analyses of Microcosm Chemistry

Soil reduction processes, as represented by the dynamics in ${NO}_{3}^{-}$, ${SO}_{4}^{2-}$, Fe(II), and PCP concentrations, were measured at regular intervals. All treatments were sampled at each time point and analyzed for the concentrations of PCP and degradation products by ultrasonic extraction and subsequent derivatization as outlined in Xu et al. (2015). Briefly, 2 g of soil sample was freeze dried and adjusted to pH 4 using 9 mM H_2_SO_4_ before being extracted with a hexane/acetone mixture (v/v, 1:1) assisted by ultrasonics for 25 min. Supernatant was separated by centrifugation and soil residue extracted twice more. Pooled supernatants were concentrated to 1 ml, combined with 0.2 mM K_2_CO_3_ before derivatization by 0.5 ml acetic anhydride. Chlorinated phenols were separated with 2 ml hexane and dehydrated using anhydrous sodium sulfate before analysis. A gas chromatograph (Agilent 6890N, Agilent, Santa Clara, CA, United States) equipped with a ^63^Ni electric capture detector (Hewlett-Packard 6890, Hewlett-Packard, Palo Alto, CA, United States) and a HP-5 MS capillary column (30 m × 0.25 mm i.d., ×0.25 μm film thickness) (J&W, Folsom, CA, United States) was used to quantify the different species of chlorophenols relative to standard controls. Temperature cycle was 80°C for 3 min, ramped at 10°C min^-1^ to 250°C, and held for 5 min. The analysis recoveries of the extraction procedures, namely the percentage of the detected PCP concentration to the initial added PCP concentration (based on 4, 8, 20, 40, and 80 μM spiked levels of PCP standard), were between 92.26 and 105.68%.

Soil pH was determined in a suspension of 1:2.5 soil/water ratio (w/v) with a pH meter (S975 SevenExcellence, Mettler-Toledo, Switzerland). Fe(II) concentration was measured using the 1,10-phenanthroline colorimetric method at 510 nm on a UV–vis spectrophotometer after extracting Fe(II) from the samples with dithionite-citrate (pH 3.0) and buffered with NaHCO_3_, in the dark (Lin et al., 2012). The determination of HCl-extractable Fe(II) was similar to the free Fe(II), except using 0.5 M HCl as substituted extractant (Xu et al., 2014) and included a range of reduced Fe(II) species, such as dissolved Fe(II), FeS, and FeCO_3_ (Heron et al., 1994). ${NH}_{4}^{+}$ was extracted at 1:10 (w/v) soil to KCl (1 mol l^-1^) ratios for 1 h at 25°C before being quantified with a continuous flow analyzer (San^++^, SKALAR, Netherlands). Nitrate and sulfate concentrations were determined by shaking 1.0 g of freeze dried soil or 2 ml soil slurry sample with 15 ml Milli-Q water for 30 min. The mixture was then centrifuged at 3000 × *g* for 10 min before being diluted 10-fold and analyzed by ion chromatography (Dionex ICS-2000, United States) equipped with an ASRS Ultra II self-regenerating suppressor as preciously described (Lin et al., 2012, 2014).

### DNA Extraction, Amplicon Amplification, and Sequencing

The microbial community of the original soil collected at day 0 (called “Original”) and soil samples collected at 40 were analyzed through amplification and sequencing of a 16S rRNA amplicon using Illumina Miseq high-throughput sequencing. The slurry sample was centrifuged for collection about 0.25 g soil and the total genomic DNA of each soil sample was extracted using FastDNA SPIN kit (Mpbio, United States) for soil according to the manufacturer’s instructions. Each DNA extract was amplified with 520F (5-AYTGGGYDTAAAGNG-3) and 802R (5-TACNVGGGTATCTAATCC-3) to obtain an approximately 250-bp fragment on the V4 region of the 16S rRNA gene. Genome DNA would be normalized to 30 ng per PCR reaction. V4 dual-index Fusion PCR Prime Cocktail and PCR Master Mix (NEB Phusion High-Fidelity PCR Master Mix) were added to the PCR run. Amplification was conducted using the PCR conditions: 30 s at 98°C, 27 cycles of 30 s at 98°C, 30 s at 50°C, and 30 s at 72°C, and a final 5 min extension at 72°C. PCR products were purified with AmpureXP beads (Agencourt) to remove the unspecific products. The final library was qualified by PicoGreen (Invitrogen, Paisley, United Kingdom). Qualified libraries were sequenced pair end on the Illumina MiSeq platform with sequencing strategy PE250 (MiSeq Reagent Kit). Illumina (Highseq2000, Illumina, San Diego, CA, United States) sequencing services were provided by the Beijing Genomics Institute (BGI, Wuhan, China).

### Sequence Analysis and Phylogenetic Classification

Fast Length Adjustment of Short Reads (FLASH) merged reads from original DNA amplicons were quality filtered following the published procedures (Caporaso et al., 2010; Magoč and Salzberg, 2011). A UPARSE pipeline (OTU clustering pipeline^1^) was used to pick operational taxonomic units (OTUs) and sequences were grouped into OTUs at 97% similarity (Edgar, 2013). A representative sequence for each OTU was selected and its identity was classified using the RDP Classifier (Wang et al., 2007). The gene sequences obtained from high-throughput analysis in this study were deposited in the NCBI sequence read archive under accession numbers SRP118766.

### Statistical Analyses

The OTU lists of each sample were submitted to the LefSe pipeline (LDA Effect size^2^) to identify significant differential features of the OTUs among treatments (Segata et al., 2011). OTU-based community diversity indice (Shannon index) of each sample was generated based on three metrics calculated by UPARSE pipeline (Edgar, 2013). The taxonomic diversity of bacteria was calculated with the phyloseq package. Statistical analyses of the experimental data were performed using the SPSS 20.0 statistical software (IBM, Armonk, IL, United States). Differences were determined by one-way analysis of variance (ANOVA) on ranks followed by Fisher’s least-significant difference. Logistic modeling was employed to examine the impacts of nitrate and sulfate on soil redox processes and PCP transformation through nonlinear curve fitting as follows (Aislabie et al., 2004; Liu et al., 2013):

$$C_{t}=\frac{a}{1+{be}^{-kt}}$$

where *t* is the incubation time (d), *C_t_* is the accumulated Fe(II) or decreased ${SO}_{4}^{2-}$/PCP concentrations at time *t*, respectively [mM for Fe(II) and ${SO}_{4}^{2-}$, μM for PCP], *a* is the maximum capacity of Fe(II) accumulation or ${SO}_{4}^{2-}$/PCP decrement, respectively, *b* is the regression coefficient, and *k* is the reaction rate constant (d^-1^). The maximum reaction rate [*V*_max_, mM d^-1^ for Fe(II) and ${SO}_{4}^{2-}$, μM d^-1^ for PCP] and the time to reach the *V*_max_ [*t*_*V*_max__, d] can be calculated from 0.25*ak* and ln*b k*^-^^1^ based on the equation.

Stoichiometric electron equivalent (eeq) analysis of the four reducing processes was carried out based on relevant half-reactions during anaerobic incubation time (Xu et al., 2015). Calculations were based on electron equivalents used for 1 mol electron acceptors of Fe(III), ${SO}_{4}^{2-}$, ${NO}_{3}^{-}$, and PCP, equating to 1, 8, 5, and 2 mol eeq, respectively.

## Results

### Dynamics of Nitrate, Sulfate, Ferrous Iron, and PCP

**FIGURE 1:**
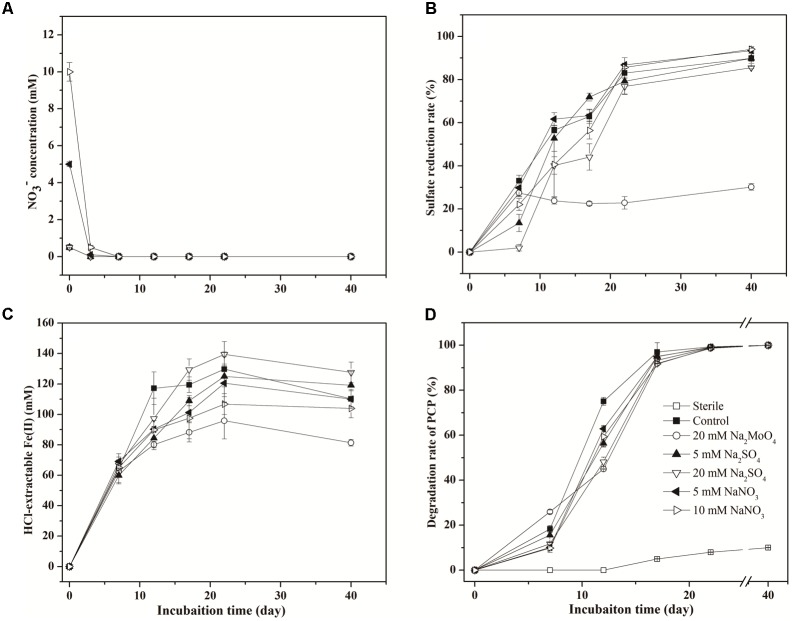
Dynamics in the concentration of ${NO}_{3}^{-}$ **(A)**, ${SO}_{4}^{2-}$ **(B)**, HCl-extractable Fe(II) **(C)**, and PCP **(D)** in different treatments.

Nitrate was depleted within 3 days in all treatments (**Figure 1A**). The sulfate reduction ratio (percentage of the decreased ${SO}_{4}^{2-}$ concentration to the initial total ${SO}_{4}^{2-}$ concentration) in the treatment with 20 mM sulfate (85.5%) was significantly lower than that in the control (89.8%) (*p* < 0.05) (**Figure 1B**), while treatment with 5 mM sulfate had no obvious difference in this ratio compared with the control. However, the *V*_max_ for sulfate reduction in treatments with sulfate was higher than that for the control, with the effect more pronounced in 20 mM than 5 mM sulfate addition (**Table 1**). The addition of nitrate exhibited no obvious effect on sulfate reduction, approaching a reduction ratio of 93.4 and 94.3% in the treatments with 5 and 10 mM nitrate, respectively (**Figure 1B** and **Table 1**). When treated with molybdate, the sulfate reduction ratio on day 7 was 27.45%, and subsequently fluctuated around 25% during incubation.

**Table 1:** The maximum reaction rate (*V*_max_) and the time to reach the *V*_max_ (*t*_*V*_max__) of ${SO}_{4}^{2-}$ and Fe(III) reduction, as well as PCP degradation in different treatments.

Calculated parameters		Treatments					
		
		Control	20 mM Na_2_MoO_4_	5 mM Na_2_SO_4_	20 mM Na_2_SO_4_	5 mM NaNO_3_	10 mM NaNO_3_
${SO}_{4}^{2-}$ reduction	*a*^a^	5.91	–^b^	9.90	23.85	6.11	6.44
	*b*^a^	9.03	–	50.71	37.91	10.37	17.52
	*k*^a^ (d^-1^)	0.21	–	0.35	0.24	0.23	0.21
	*V*_max_ (mM d^-1^)	0.31	–	0.87	1.43	0.35	0.34
	*t*_*V*_max__ (d)	10.48	–	11.22	15.15	10.17	13.63
	*R*^2^	0.98	–	0.99	0.97	0.98	0.99
Fe(III) reduction	*a*	119.74	86.85	120.76	133.17	109.36	101.27
	*b*	179.09	37.79	10.09	12.14	11.78	19.21
	*k* (d^-1^)	0.76	0.65	0.29	0.32	0.40	0.49
	*V*_max_ (mM d^-1^)	22.75	14.11	8.76	10.65	10.94	12.41
	*t*_*V*_max__ (d)	6.83	5.59	7.97	7.80	6.17	6.03
	*R*^2^	0.99	0.99	0.99	0.99	0.98	0.99
PCP degradation	*a*	149.61	146.70	151.48	151.42	149.36	148.63
	*b*	170.26	38.71	120.02	225.02	364.05	262.50
	*k* (d^-1^)	0.52	0.31	0.42	0.45	0.54	0.50
	V_max_ (μM d^-1^)	19.45	11.37	15.91	17.03	20.16	18.58
	*t*_*V*_max__ (d)	9.88	11.79	11.40	12.04	10.92	11.14
	*R*^2^	1.00	0.99	1.00	1.00	1.00	1.00


*^*a*^The parameter values for model variables represent best-fit values. ^*b*^Not suitable to be calculated with the tested modeling.*

Concentrations of HCl-extractable Fe(II) followed similar variation trends in all treatments, increasing markedly during the first 7 days (**Figure 1C**). Slight differences between treatments appeared after 7 days and proceeded to the end of incubation, with the concentration of HCl-extractable Fe(II) lower in treatments with nitrate than those with sulfate. However, the impacts of sulfate and nitrate on the Fe(III) reduction were more noticeable as shown by logistic modeling analysis (**Table 1**). The values of *V*_max_ were 8.76, 10.65, 10.94, and 12.41 mM d^-1^ in the treatments with 5 mM sulfate, 20 mM sulfate, 5 mM nitrate, and 10 mM nitrate, respectively, and that for the control was 22.75 mM d^-1^. This suggested that addition of nitrate and sulfate both inhibited the Fe(III) reduction. And interestingly, addition of nitrate did not delay the *t*_*V*_max__ for Fe(III) reduction compared to those in the control, while sulfate did. Correspondingly, the lowest concentration of HCl-extractable Fe(II) was exhibited in the treatment with molybdate, recording the lowest Fe(II) production after 7 days’ incubation (**Figure 1C**).

In the sterile treatment, PCP transformation was minimal (<10%) (**Figure 1D**), indicating that the decrease of PCP through abiotic process or sorption was negligible within the microcosms. As for the other non-sterile treatments, in the first 12 days, the PCP degradation was significantly inhibited following sulfate addition, compared to the control (75.1%). Degradation rates of 56.4% (*p* < 0.05) and 48.2% (*p* < 0.05) in 5 mM and 20 mM sulfate treatments were observed, respectively. PCP degradation also decreased to 62.9 and 59.4% with the addition of 5 and 10 mM nitrate, respectively (*p* < 0.05). The values of *V*_max_ and *t*_*V*_max__ during PCP degradation in **Table 1** showed similar inhibition influences of sulfate and nitrate on reductive dechlorination of PCP, with the effect more significant following sulfate addition by comparison with nitrate addition. However, on day 22, differences in the PCP degradation rates were no longer apparent between the control and treatments containing either nitrate or sulfate (**Figure 1D**). This suggested that both sulfate and nitrate addition inhibited the reductive dechlorination of PCP, but their inhibition effect lessened over time with no obvious difference at the end of incubation.

### Anaerobic Transformation of PCP

**FIGURE 2:**
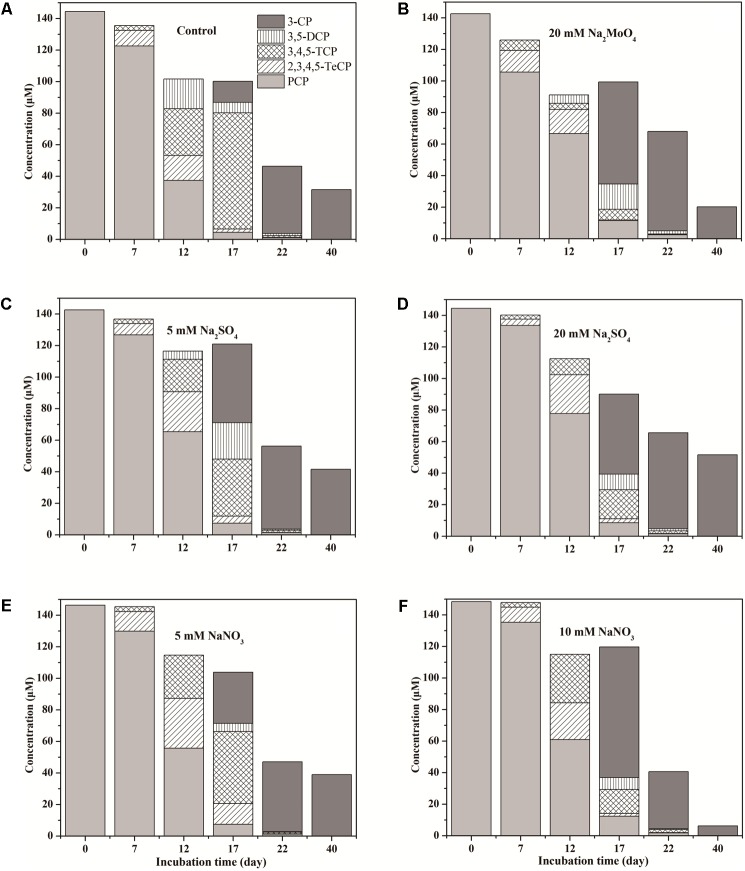
Dynamics of PCP dechlorinated metabolites in treatments of Control **(A)**, 20 mM Na_2_MoO_4_ **(B)**, 5 mM Na_2_SO_4_ **(C)**, 20 mM Na_2_SO_4_ **(D)**, 5 mM NaNO_3_ **(E)**, and 10 mM NaNO_3_ **(F)**.

The anaerobic transformation pathway of PCP was studied by detecting degradation metabolites in all treatments during the 40 days incubation (**Figure 2**). Dechlorinated metabolites, including TeCP, TCP, DCP, and 3-CP with one to four chlorines dechlorinated were detected in all treatments except sterilized control. No phenol and other isomeride dechlorinated metabolites were detected. The metabolites 2,3,4,5-TeCP and 3,4,5-TCP were detected in the first 7 days. On day 17, 3,4,5-TCP and 3-CP were detected as the major intermediate product, and little residual PCP was detected (varying from 4.49 to 12.39 μM). At the end of incubation, the 3-CP was the major end product of PCP degradation. As a result, PCP in our tested soil might be transformed through the pathway PCP → 2,3,4,5-TeCP → 3,4,5-TCP → 3,5-DCP → 3-CP during anaerobic incubation.

### Microbial Community Composition

After quality control, 112022–155268 reads were retrieved after Illumina Miseq sequencing. The average OTU numbers ranged from 4349 to 4553 at 97% similarity across all treatments (**Table 2**). Shannon indexes in samples of 5 and 10 mM nitrate were 6.53 and 6.44, respectively. Moreover, the Shannon indexes were 6.51 and 6.24 with 5 and 20 mM sulfate, respectively. The Shannon indexes indicated a slight decrease in the bacterial diversity in high concentration of nitrate (10 mM) or sulfate (20 mM) when compared to the control (6.46).

**Table 2:** Community richness and diversity indices for the soil samples of different treatments.

Treatment^a^	OTU	Shannon index
Original soil	4362	6.32
Control	4407	6.46
20 mM Na_2_MoO_4_	4349	6.38
5 mM Na_2_SO_4_	4553	6.51
20 mM Na_2_SO_4_	4378	6.24
5 mM NaNO_3_	4396	6.53
10 mM NaNO_3_	4390	6.44


*^*a*^Original soil, sample was on day 0; other treatments, samples were on day 40.*

**FIGURE 3:**
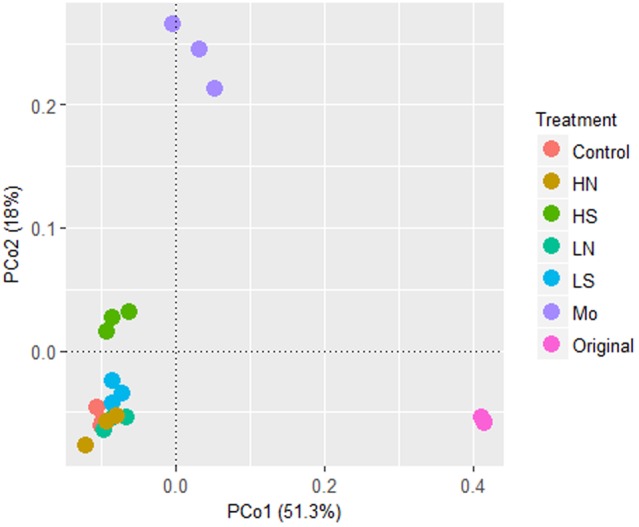
PCoA analysis based on the relative abundances of bacterial pyrotag sequences. Symbols: Control, treatment without addition; HN, 10 mM NaNO_3_; HS, 20 mM Na_2_SO_4_; LN, 5 mM NaNO_3_; LS, 5 mM Na_2_SO_4_; Mo, 20 mM Na_2_MoO_4_; Original, the original soil at day 0. Three replicates were conducted among one treatment.

Differences in bacterial community structures induced by different treatments were visualized by PCoA analysis (**Figure 3**). The separation was mainly explained by the PC1 with 51.3% of variance, and the PC2 with 18% of variance. After 40 days anaerobic incubation, the microbial community structure differentiated in the treatments compared to the original soil microbial community structure (*p* < 0.001). The bacterial communities in treatments with nitrate and 5 mM sulfate were clustered together, but differed in treatments with 20 mM sulfate and 20 mM molybdate, respectively, indicating that high sulfate concentration had a more significant influence on microbial community structures than nitrate.

**FIGURE 4:**
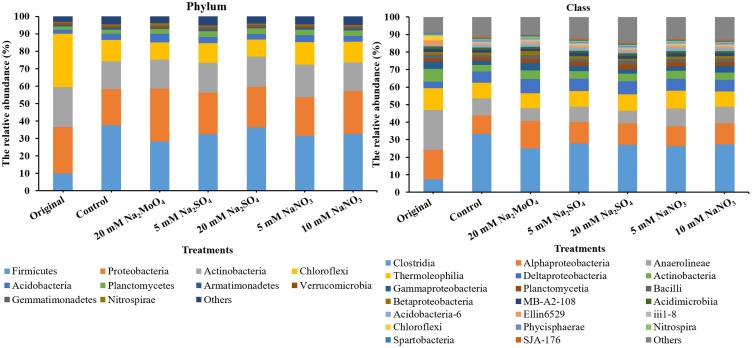
The bacterial community structure at the phylum and class level. Original, soil sample was on day 0 and Other treatments, samples were on day 40.

The top 4 phyla in all treatments were Firmicutes, Proteobacteria, Actinobacteria, and Chloroflexi, accounting for more than 85% of the reads (**Figure 4**). In total, the relative abundance of Firmicutes increased while that of Chloroflexi decreased after incubation. At the class level, the microbial community was dominated by Clostridia in all treatments with a majority of other sequences being grouped into Alphaproteobacteria, Anaerolineae, Deltaproteobacteria, Thermoleophilia, Actinobacteria, and Gammaproteobacteria (**Figure 4**). Compared to the control, the relative abundance of Clostridia and Anaerolineae significantly decreased in treatments with nitrate and sulfate addition (on an average, decreased from 33.4 to 27.1% and decreased from 9.9 to 8.8%, respectively, *p* < 0.05). Conversely, the relative abundance of Alphaproteobacteria, Deltaproteobacteria, Actinobacteria, Planctomycetia, and Bacilli increased in all the treatments (*p* < 0.05). Particularly, the relative abundance of group Acidobacteria increased in the treatments with nitrate addition but decreased with sulfate addition.

**FIGURE 5:**
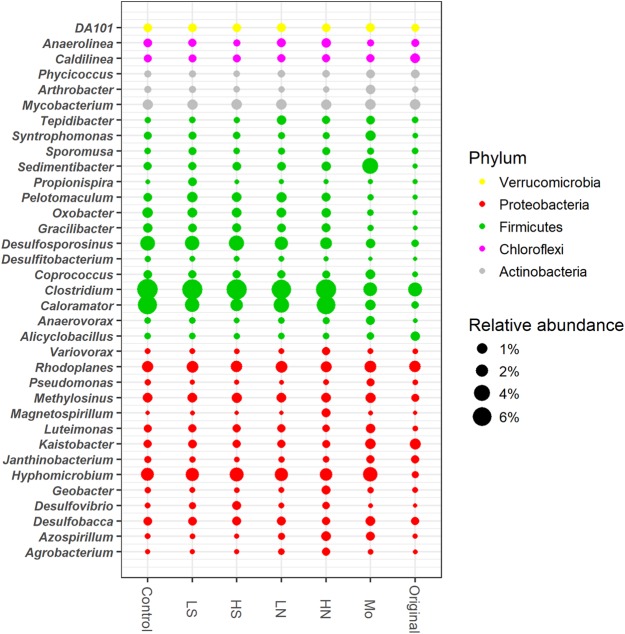
Dominant bacterial genera in all treatments. Original, soil sample was on day 0. Other treatments, samples were on day 40. The meanings of the abbreviations in the graph are the same as in **Figure 3**.

Dominant genera were mainly affiliated to Firmicutes and Proteobacteria, including the members of *Clostridium*, *Desulfosporosinus*, *Caloramator*, *Desulfobacca*, *Hyphomicrobium*, *Rhodoplanes*, *Pelotomaculum*, *Geobacter*, *Desulfitobacterium*, *Desulfovibrio*, and *Sedimentibacter* (**Figure 5**). Compared to the original day 0, the relative abundance of *Hyphomicrobium*, *Clostridium*, *Caloramator*, *Desulfosporosinus*, *Oxobacter*, and *Gracilibacter* in sulfate and nitrate treatments significantly increased after incubation. The relative abundances of most genera were lower in the treatment with molybdate than those with nitrate or sulfate, in particular for members of *Clostridium*, *Caloramator*, *Desulfovibrio*, *Pelotomaculum*, *Oxobacter*, and *Desulfosporosinus*. The relative abundance of *Desulfitobacterium* was below 1% in all treatments with sulfate and nitrate and was lower than that in the control but relatively higher than that in the day 0. The relative abundance of *Desulfovibrio* increased consistently following sulfate addition, with the difference reached significant in 20 mM sulfate treatment compared to that in 5 mM sulfate and control treatments (*p* < 0.05). The relative abundances of *Clostridium*, *Azospirillum*, and *Caloramator* increased with increased nitrate concentrations, while sulfate addition had no effect on the growth of these members as evidenced by their relative abundances.

## Discussion

### The Effect of Sulfate and Nitrate on the Dechlorination of PCP

Our study conducted microcosm experiments to explore the effect of sulfate and nitrate on the degradation of PCP in the paddy soil using lactate as metabolic substrate. The anaerobic dechlorination ratios of PCP in all treatments in the first 7 days were slow. The lag time of 3–7 days was observed across all microcosms prior to dechlorination of PCP and likely due to limiting environmental conditions (e.g., soil nutrients, trace minerals, and electron donors) as reported in previous studies (Chen et al., 2012; Liu et al., 2013). Both ${NO}_{3}^{-}$ and ${SO}_{4}^{2-}$ have been reported to inhibit the reductive dechlorination of PCP in a paddy soil (Lin et al., 2014); however, other studies have also reported that PCP dechlorination was enhanced under sulfate reduction and inhibited during denitrification over 20-day incubation periods (Chang et al., 1996). In our study, dynamic processes combined with logistic model simulation indicated that both sulfate and nitrate were found to inhibit the reductive dechlorination of PCP and Fe(III) reduction (**Figure 1** and **Table 1**) even with different inhibition effect.

Although nitrate in most treatments was reduced within 3 days, it still had profound effects on the reduction of Fe(III) and PCP. Generally, nitrate is the first electron acceptor to be reduced once oxygen is depleted in paddy soils before Fe(III). In our study, the higher the concentration of nitrate, the lower the ratio of Fe(III) reduction (**Figure 1C**). This indicated the presence of nitrate inhibited Fe(III) reduction where nitrate and iron oxides coexisted and was similar to the previous studies (Cooper et al., 2003; Matocha and Coyne, 2007). Meanwhile, Fe(III) was reduced more quickly in the treatment with 20 mM sulfate than that with 5 mM sulfate (**Figure 1C**). Previous studies showed that the reduction of Fe(III) can be slowed and limited in the presence of a low sulfate concentration (0.2 mM), but increased >10 times with high sulfate amendment (10.2 mM) due to the increased biogenic-sulfide-driven-Fe(II) production (Kwon et al., 2014). Therefore, the Fe(III) reduction was significantly influenced mainly because that the nitrate and sulfate addition changed the availability of Fe(III) in our study.

Compared to nitrate, the addition of sulfate greatly inhibited PCP dechlorination since the value of *V*_max_ was relatively lower and *t*_*V*_max__ was much higher when compared to these in the control (**Table 1**). Previous researches have proposed that electron acceptors (nitrate and sulfate) inhibited the degradation of organic pollutants due to competition for electron donors (Heimann et al., 2005; Aulenta et al., 2007; Chen et al., 2012). Lactate has been previously demonstrated to be an effective fermented electron donor for respiration by a wide range of microorganisms during the transformation of organic pollutants (Freeborn et al., 2005; Thomas and Gohil, 2011). From the balance of the electron equivalent (eeq) used in the incubation, most electrons transferred to the reduction of Fe(III) (1.22–1.91 mmol), sulfate (0.24–2.84 mmol), and nitrate (0.375–0.75 mmol), while PCP reductive dechlorination (0.02 mmol) was a minor pathway for electron flow, accounting for only 5‰ of the total consumed electron equivalents (3.6 mmol) (**Table 3**). Our previous research found that supplying excess electron donors may not necessarily achieve substantial dechlorination (Xue et al., 2017). The rapid fermentation of lactate may also result in the transient build-up of H_2_ to levels around two orders of magnitude higher compared to the steady-state conditions (Heimann et al., 2005). Hydrogen supplied by hollow-fiber membranes, maintaining adequate hydrogen above hydrogen thresholds for dechlorination, also inhibited dechlorination activity even when hydrogen was not limiting in the presence of nitrate and sulfate (Freeborn et al., 2005). Hence, competition for electron donor is not solely responsible for the inhibition of dechlorination in the presence of sulfate and nitrate, and the microbial interactions were thus predicted to have significant effects on these redox processes.

**Table 3:** Balance of the electron equivalents used for Fe(III)/${SO}_{4}^{2-}$/${NO}_{3}^{-}$ reduction and PCP dechlorination at the end of incubation.

Treatment	Electron equivalents (eeq, mmol) used for					
	
	Eeq^a^ added	Fe(III) reduction	${NO}_{3}^{-}$ reduction	${SO}_{4}^{2-}$ reduction	Dechlorination	Eeq consumed
Control	3.60	1.65	0	0.72	0.021	2.391
20 Mm Na_2_MoO_4_	3.60	1.22	0	0.24	0.021	1.481
5 Mm Na_2_SO_4_	3.60	1.78	0	1.26	0.020	3.060
20 Mm Na_2_SO_4_	3.60	1.91	0	2.84	0.020	4.770
5 Mm NaNO_3_	3.60	1.65	0.375	0.75	0.021	2.796
10 Mm NaNO_3_	3.60	1.56	0.75	0.76	0.022	3.092


*^*a*^Eeq, electron equivalents.*

### The Pathway of PCP Degradation

The vital step in the biodegradation of PCP is the removal of the chlorine atoms. Previous studies reported that PCP under anaerobic conditions first undergoes *ortho*-dechlorination (Susarla et al., 1997; Villemur et al., 2006; Zhang et al., 2012). Two pathways for PCP degradation have been proposed (Susarla et al., 1997). Under sulfate-reducing condition, PCP can be transformed through the pathway PCP → 2,3,5,6-TeCP → 2,3,5-TCP → 3,5-DCP → 3-CP. Under methanogenic condition, PCP may transform through the pathway PCP → 2,3,4,5-TeCP → 3,4,5-TCP → 3,4-DCP → 3-CP. In our study, PCP was transformed through the pathway PCP → 2,3,4,5-TeCP → 3,4,5-TCP → 3,5-DCP → 3-CP in all treatments. Bacteria such as *Desulfitobacterium dehalogenans*, *Desulfitobacterium chlororespirans*, *Desulfitobacterium hafniense*, and *Desulfitobacterium* sp. strain PCE1 preferentially remove the chlorine atom at the *ortho*-position of PCP rather than the *meta*- or *para*-positions (Dennie et al., 1998). Most *Desulfitobacterium* strains have been reported to play an important role in the degradation of halogenated organic compounds such as tetrachloroethene, trichloroethene, and carbon tetrachloride (Gerritse et al., 1996; Villemur et al., 2006; Bisaillon et al., 2010; Zhao et al., 2015), but very few have been studied for degradation of PCP, likely due to the toxicity of PCP to these bacteria. *D. hafniense* strain PCP-1 (formerly *frappieri* PCP-1) is the known strict anaerobic bacterium which has been proved capable of dechlorinating PCP at the *ortho-*, *para-*, and *meta-*position (Dennie et al., 1998; Bisaillon et al., 2010). The sequence of dechlorination was the same as our findings including no dechlorinated metabolites beyond 3-CP. *D. hafniense* strain PCP-1 was reported to have several reductive dehalogenase (RDase) genes that could carry out the sequential reductive dechlorination of PCP; and CprA3 reductive dehalogenase showed high *ortho-*dechlorination activity toward PCP (Bisaillon et al., 2010). In our study, members of *Desulfitobacterium* have been detected and the relative abundance was enriched during incubation with PCP stress. Therefore, the *Desulfitobacterium* is potentially an essential to PCP dechlorination in the tested soil.

### Microbial Community Structure During Competitive Microbial Dechlorination of PCP in the Presence of Nitrate and Sulfate

The predominant genera *Desulfovibrio*, *Desulfosporosinus*, *Geobacter*, *Desulfobacca*, *Hyphomicrobium*, *Pelotomaculum*, *Sedimentibacter*, *Mycobacterium*, *Caloramator*, *Rhodoplanes*, and *Clostridium* were detected in the treatment with both nitrate and sulfate (**Figure 5**). The presence of such genera across all the microcosms and during dechlorination of PCP indicated that they were tolerant to PCP and the associated degradation products. The role, directly or indirectly, of this core group during PCP transformation in all treatments was of interest. The mentioned genera have been reported to have members capable of facilitating the dechlorination of chlorinated organic pollutants. For example, the genera *Desulfovibrio* and *Clostridium* were reported to have the ability of using lactate or acetate to generate H_2_ serving as an electron donor in a dechlorinating consortium (Freeborn et al., 2005; Behrens et al., 2008). *Clostridium* has also been previously reported as one of the common microbial community members during PCP degradation (Tartakovsky et al., 2001). Thus, the potential interaction or competition between these above mentioned versatile or PCP tolerate groups and the known dechlorinators (e.g., *Desulfitobacterium*) would happen, which were considered as the cause of suppressed PCP dechlorination by sulfate or nitrate.

Compared with nitrate, sulfate addition imposed more severe inhibition effect on PCP dechlorination at the early stage of incubation (**Figure 1** and **Table 1**). Previous studies have shown that the reductive dechlorinating bacteria and sulfate-reducing bacteria often share biotopes in soils contaminated with chlorinated compounds (Drzyzga et al., 2001), which caused competition for available nutrients and other resources in the limited environment. This resulted in complicated interactions between and within the functional dechlorinators and sulfate-reducers inhabiting a common habitat. As mentioned before, indigenous carbon sources used in this study were adequate for the dechlorination of PCP and other redox processes (**Table 3**), therefore, the inhibition due to the limitation of resources is expected to be negligible in sulfate added treatments.

**FIGURE 6:**
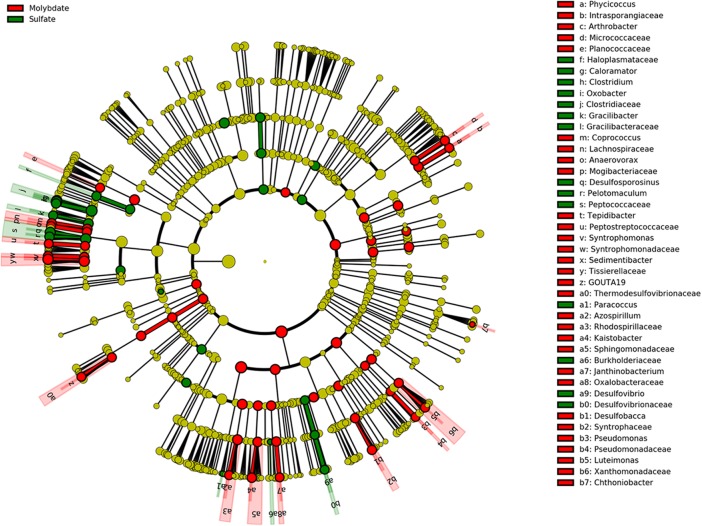
Cladograms indicating the phylogenetic distribution of bacteria lineages associated with sulfate and molybdate addition. The phylum, class, order, family, and genus levels are listed in order from inside to outside of the cladogram and the labels for levels of family and genus are abbreviated by single letter. The green and red circles represent the bacteria with differences reached significant in the treatment with sulfate and molybdate addition, respectively; whereas the yellow circles represent the taxa with no significant differences between both treatments.

Molybdate was used as an inhibitor to estimate whether or not sulfate reducers could make a difference on PCP dechlorination, since it has the ability to inhibit the key enzyme of ATP sulfurylase in the pathway of sulfate reduction (Ranade et al., 1999). The sulfate reduction was greatly inhibited by molybdate addition, as well as PCP transformation (**Figures 1B,D**). A cladogram produced by LefSe highlighted the noticeable bacterial members that differed between sulfate and molybdate treatments (**Figure 6**). Results demonstrated that genera of *Caloramator*, *Clostridium*, *Oxobacter*, *Gracilibacter*, *Desulfosporosinus*, *Pelotomaculum*, *Paracoccus*, and *Desulfovibrio* exhibited significant variation following sulfate addition. Microbial analysis revealed a substantial decrease of most genera in molybdate treatments, especially for the known sulfate reducers, such as *Desulfovibrio* and *Desulfosporosinus* (**Figure 6**). Relatively, the *Desulfovibrio* was enriched in the treatments with sulfate but not abundant in the treatments with nitrate or the control (**Figure 5**). We can thus speculate that the addition of sulfate might stimulate the growth of *Desulfovibrio*.

Previous studies have shown that sulfate-reducing bacteria such as *Desulfovibrio vulgaris*, *Desulfovibrio gigas*, and *Desulfovibrio desulfuricans* are able to metabolize lactate or H_2_ when grown in the absence of sulfate or in the media with low sulfate concentrations (Cabirol et al., 1998). Through syntrophic association with group of *Desulfovibrio*, dechlorinators such as *Desulfitobacteria* may acquire their electrons by interspecies hydrogen and acetate transfer (Cabirol et al., 1998). Syntrophy between the sulfate reducing bacteria *Desulfovibrio* and the dehalorespiring bacteria *Desulfitobacterium* via interspecies H_2_ transfer occurred only at low concentrations or in the absence of sulfate, while in the high sulfate concentration environment (>2.5 mM), dechlorinating bacteria was outnumbered by sulfate reducer of *Desulfovibrio*, and dehalogenation was not occurring (Drzyzga et al., 2001; Drzyzga and Gottschal, 2002; Maphosa et al., 2010). Thus, the inhibition effect of PCP dechlorination by sulfate should be ultimately ascribed to the sulfate reducers of *Desulfovibrio* to outcompete dechlorinators under high sulfate concentration, while deficient sulfate levels (e.g., the control) facilitated PCP transformation because of the syntrophic relationship between sulfate reducer of *Desulfovibrio* and dehalorespiring bacteria of *Desulfitobacterium*. In addition, the inhibition of sulfate and nitrate on reductive dechlorination of PCP in this study gradually lessened during the 40-day incubation, since the decrease in concentration of sulfate and nitrate would favor the growth of dechlorinating bacteria and thereby PCP transformation.

By coupling the typical soil redox processes with the response of the microbial community structure during PCP degradation in natural flooded paddy soils, this study improved the understanding regarding the microbial competitive dechlorination of PCP during nitrate and sulfate reduction. Increased sulfate and nitrate reduction inhibited the process of PCP dechlorination. PCP transformation started from *ortho-*position, then dechlorinate at *para-* and *meta-*position to form 3-CP as the final product. Analyses for the microbial community structures revealed that although soil bacterial community structure shared similar dominate species following addition of nitrate and sulfate, respectively, some specialized functional species were also responded contrastingly to the addition of sulfate and nitrate, with the genus of *Desulfovibrio* enriched in the treatments with sulfate individually, thereby mediated a different competitive microbial dechlorination of PCP. Overall, our results suggest that a shared existence of electron acceptors, such as sulfate and nitrate, could change the microbial diversity by allowing bacteria with special metabolic capabilities to grow in the soil and sediment polluted with PCP. This is crucial for understanding the self-purification function of paddy soils once they are polluted by PCP, under the condition of excessive use of nitrogen fertilizer as well as accumulation of iron sulfur minerals. Furthermore, besides nitrate and sulfate, iron plays a particularly important role in environmental biogeochemistry. Hence, the effect of Fe(III) reduction on the dechlorination of chlorinated compounds, through either biotic or abiotic way, will deserve to study in the future.

## Author Contributions

YX and LX were the main contributors to perform the experiments, analyze the data and write the manuscript. YH and JX designed the work. QY and XF participated in the experiments. MZ and QY participated in the data analysis. AF and YH carefully revised the manuscript.

## Conflict of Interest Statement

The authors declare that the research was conducted in the absence of any commercial or financial relationships that could be construed as a potential conflict of interest.

## Footnotes

1 http://drive5.com/uparse/

2 http://huttenhower.sph.harvard.edu/galaxy
